# Supplementary material for: Phyloecology of nitrate ammonifiers and their importance relative to denitrifiers in global terrestrial biomes
Source: Nat Commun. 2023 Dec 12;14:8249. doi: 10.1038/s41467-023-44022-3 (PMC10716430; doi:10.1038/s41467-023-44022-3)
Supplement: Supplementary file 1 — Supplementary information [file 41467_2023_44022_MOESM1_ESM.pdf]

**Supplementary information**

# Phyloecology of nitrate ammonifiers and their importance relative to denitrifiers in global terrestrial biomes

Aurélien Saghaï, Grace Pold, Christopher M. Jones and Sara Hallin

**Content:**

Supplementary Tables 1-4

Supplementary Figures 1-10

**Supplementary Table 1.** Phylum distribution of the assemblies carrying *nrfA* and/or *onr* included in this study.

| Phylum | Nb. *nrfA*-assemblies | Nb. *onr*-assemblies | Nb. *nrfA*- and *onr*-assemblies |
|---|---|---|---|
| AABM5-125-24 | 2 | | |
| Acidobacteriota | 51 | 1 | 1 |
| Actinobacteriota | 78 | 4 | |
| Armatimonadota | 11 | 3 | |
| Bacteroidota | 287 | 3 | |
| Bdellovibrionota | 9 | | |
| Calditrichota | 1 | | |
| Campylobacterota | 51 | | |
| Chloroflexota | 61 | 1 | |
| Chrysiogenetota | 2 | | |
| CLD3 | 2 | | |
| Cyanobacteria | 2 | | |
| Deferribacterota | | 3 | |
| Deinococcota | 7 | | |
| Desulfobacterota | 118 | 34 | 9 |
| Elusimicrobiota | 4 | | |
| Eremiobacterota | 1 | | |
| FEN-1099 | 1 | | |
| Fibrobacterota | 1 | | |
| Firmicutes | 182 | 1 | 1 |
| Gemmatimonadota | 6 | | |
| Goldbacteria | 1 | | |
| Hydrogenedentota | 6 | | |
| Krumholzibacteriota | 3 | | |
| KSB1 | 3 | | |
| Latescibacterota | 1 | | |
| Margulisbacteria | | 1 | |
| Marinisomatota | 1 | | |
| Methylomirabilota | 2 | | |
| Myxococcota | 37 | | |
| Nitrospinota | 2 | | |
| Nitrospirota | 14 | 13 | 1 |
| OLB16 | 1 | | |
| Omnitrophota | 1 | | |
| Planctomycetota | 31 | 2 | |
| Poribacteria | 1 | | |
| Proteobacteria | 88 | 23 | |
| QNDG01 | 1 | | |
| Schekmanbacteria | | 1 | |
| Spirochaetota | 12 | | |
| Sumerlaeota | 4 | | |
| UBA10199 | 3 | | |
| UBP17 | 1 | | |
| UBP4 | 1 | | |
| Verrucomicrobiota | 15 | 1 | |
| Zixibacteria | 2 | | |
| | | | |
| Halobacteriota | 5 | | |
| Hydrothermarchaeota | | 2 | |

**Supplementary Table 2.** Distribution of *nir*, *nor* and *nosZ* genes in the assemblies harboring *nrfA* and *onr*.

| | Gene or gene combination | Number of assemblies |
|---|---|---|
| *nrfA*-assemblies (n = 1,113) | *nrfA* only | 647 |
| | *nir* only | 63 |
| | *nor* only | 161 |
| | *nosZ* only | 93 |
| | *nir* + *nor* | 64 |
| | *nir* + *nosZ* | 11 |
| | *nor* + *nosZ* | 46 |
| | *nir* + *nor* + *nosZ* | 28 |
| *nrfA*-assemblies in CXXCH clade (n = 310) | *nrfA* only | 125 |
| | *nir* only | 32 |
| | *nor* only | 60 |
| | *nosZ* only | 27 |
| | *nir* + *nor* | 35 |
| | *nir* + *nosZ* | 7 |
| | *nor* + *nosZ* | 17 |
| | *nir* + *nor* + *nosZ* | 7 |
| *onr*-assemblies (n = 93) | *onr only* | 56 |
| | *nor only* | 27 |
| | *nir* + *nor* | 5 |
| | *nor* + *nosZ* | 2 |
| | *nir* + *nor* + *nosZ* | 3 |
| *nrfA*- and *onr*-assemblies (n = 12) | *nrfA* and *onr* only | 4 |
| | *nor* only | 8 |

**Supplementary Table 3.** Biome identity, number and reference of soil metagenomes included in this study. Unpublished metagenomes are indexed with their NCBI BioProject number (PRJNA).

| Biome | Nb. of metagenomes | Reference |
|---|---|---|
| Croplands | 41 | Ref. 1 |
| | 25 | Ref. 2 |
| | 3 | Ref. 3 |
| | 12 | Ref. 4 |
| | 36 | PRJNA717057 |
| | 20 | Ref. 5 |
| Deserts and Xeric Shrublands | 3 | Ref. 6 |
| | 39 | Ref. 1 |
| | 65 | NEON* |
| Boreal Forests & Taiga | 21 | Ref. 7 |
| | 7 | Ref. 6 |
| | 54 | NEON* |
| Mediterranean Forests Woodlands and Scrub | 17 | Ref. 6 |
| | 45 | Ref. 1 |
| | 22 | NEON* |
| Temperate Broadleaf and Mixed Forests | 21 | Ref. 7 |
| | 66 | Ref. 6 |
| | 139 | Ref. 1 |
| | 244 | NEON* |
| | 12 | Ref. 8 |
| | 11 | PRJNA717057 |
| Temperate Conifer Forests | 44 | Ref. 7 |
| | 8 | Ref. 6 |
| | 59 | Ref. 9 |
| | 152 | NEON* |
| Tropical and Subtropical Dry Broadleaf Forests | 24 | NEON* |
| Tropical and Subtropical Moist Broadleaf Forests | 78 | Ref. 6 |
| | 6 | Ref. 1 |
| | 2 | Ref. 3 |
| Temperate Grasslands Savannas and Shrublands | 2 | Ref. 6 |
| | 12 | Ref. 1 |
| | 169 | NEON* |
| Tropical and Subtropical Grasslands Savannas and Shrublands | 13 | Ref. 6 |
| | 29 | Ref. 1 |
| Tundra | 5 | Ref. 6 |
| | 27 | NEON* |
| | 56 | Ref. 10 |

*NEON (National Ecological Observatory Network). Soil microbe metagenome sequences (DP1.10107.001), RELEASE-2021. https://doi.org/10.48443/fzzj-g053.

**Supplementary Table 4.** Plant host identity, number and reference of rhizosphere metagenomes included in this study. Unpublished metagenomes are indexed with their NCBI BioProject number (PRJNA).

| Plant host species | Nb. of metagenomes | Reference |
|---|---|---|
| *Amaranthus* sp. | 13 | Ref. 11 |
| *Arabidopsis thaliana* | 49 | Ref. 12 |
| *Asparagus* sp. | 12 | Ref. 13 |
| *Phaseolus vulgaris* | 22 | Ref. 3 |
| *Brassica alboglabra* | 16 | Ref. 11 |
| *Brassica parachinensis* | 15 | Ref. 11 |
| *Citrus* sp. | 23 | Ref. 5 |
| *Colobanthus quitensis* | 3 | Ref. 14 |
| *Colobanthus quitensis + Deschampsia antarctica* | 3 | Ref. 14 |
| *Zea mays* | 32 | PRJNA330341-47, PRJNA367156-68, PRJNA405457, PRJNA406023-27, PRJNA444376-80 |
| *Gossypium* sp. | 1 | Ref. 15 |
| *Miscanthus* sp. | 43 | PRJNA330359-60, PRJNA365493-99, PRJNA366147-53, PRJNA366178-79, PRJNA367152 -53, PRJNA375575-80, PRJNA405458-61, PRJNA444381-85 |
| *Populus* sp. | 13 | Ref. 16 |
| *Helianthus annuus* | 1 | Ref. 17 |
| *Panicum virgatum* | 25 | PRJNA330352-58, PRJNA365487-92, PRJNA375569-74, PRJNA405463-67, PRJNA444386 |
| *Taxus cuspidata* | 1 | PRJNA418191 |

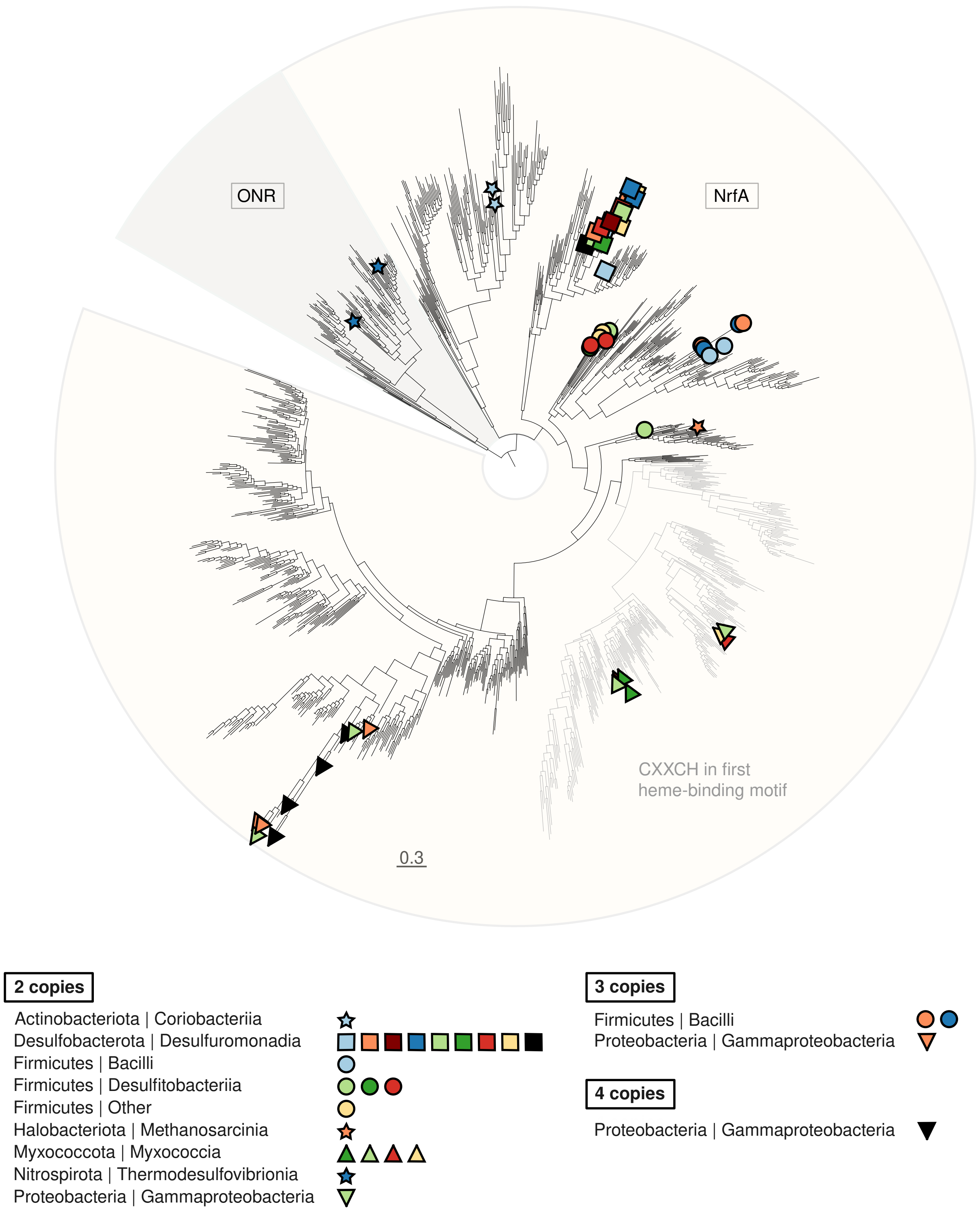


**Supplementary Figure 1.** Maximum likelihood phylogeny of full-length NrfA and ONR sequences showing the presence of multiple *nrfA* and *onr* copies in genome assemblies. Taxonomic classification at the phylum and class level of the most abundant classes in the phylogeny (see Fig. 1) is indicated and is based on the Genome Taxonomy DataBase. Each unique combination of symbol/color correspond to one assembly. The scale bar denotes the amino acid exchange rate (WAG+R10). The tree was inferred from the alignment of 350 amino acid positions.

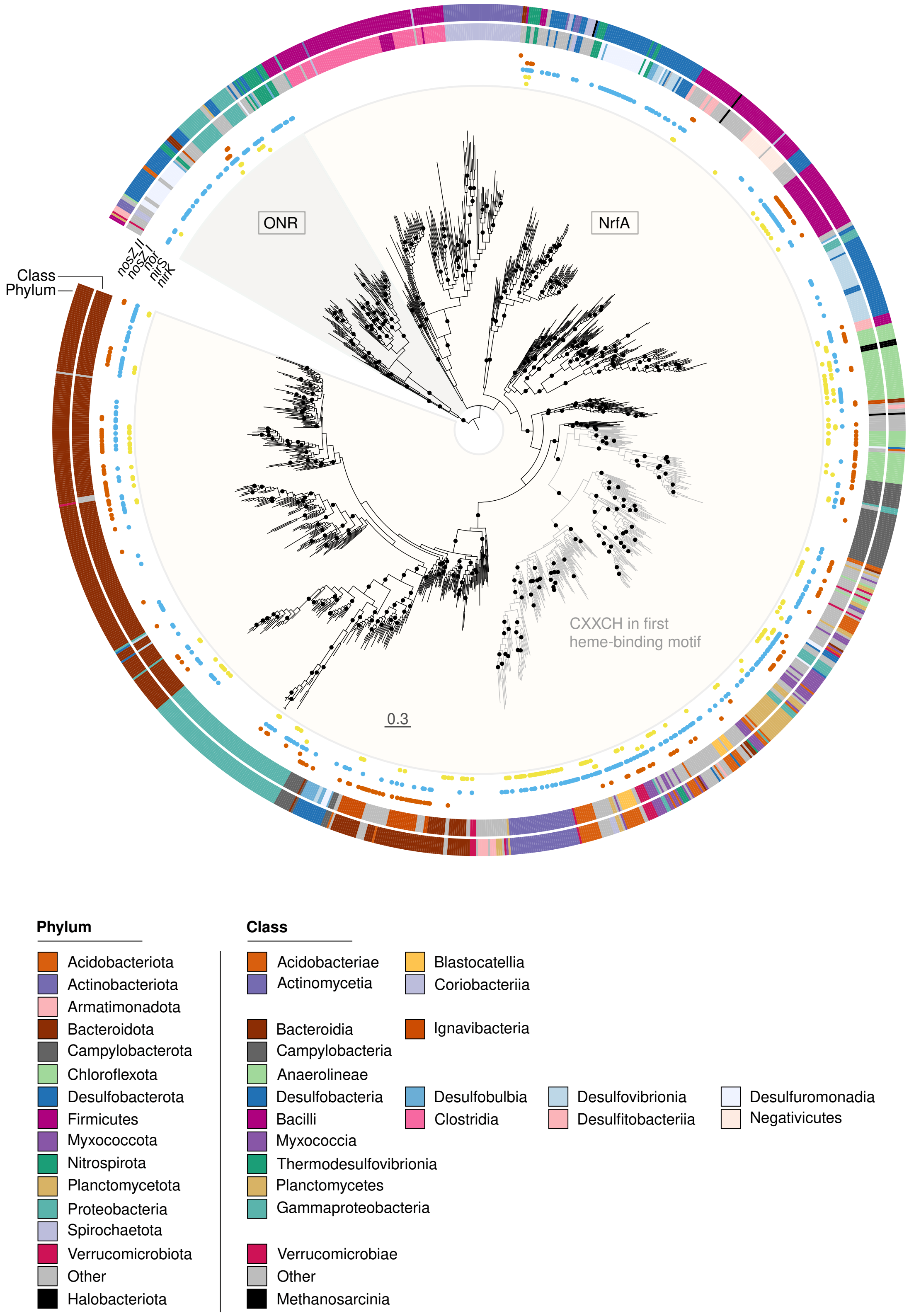


**Supplementary Figure 2. Maximum likelihood phylogeny of full-length NrfA and ONR sequences showing the co-existence of *nrfA* and *onr* with denitrification genes in genome assemblies.** The presence of *nirK* (yellow), *nirS* (yellow), *nor* (blue) and *nosZ* (clade I and II; red) in the assemblies corresponding to each NrfA/ONR sequence is indicated in the inner rings. Taxonomic classification at the phylum and class level of the most abundant classes (n > 10, except for the archaeal class Methanosarcinia) is indicated by the color in the two outer rings and is based on the Genome Taxonomy DataBase. Black circles on the phylogeny show support values (SH-aLRT test ≥ 80 % and ultrafast bootstrap ≥ 95 %) and the scale bar denotes the amino-acid exchange rate (WAG+R10). The tree was inferred from the alignment of 350 amino acid positions.

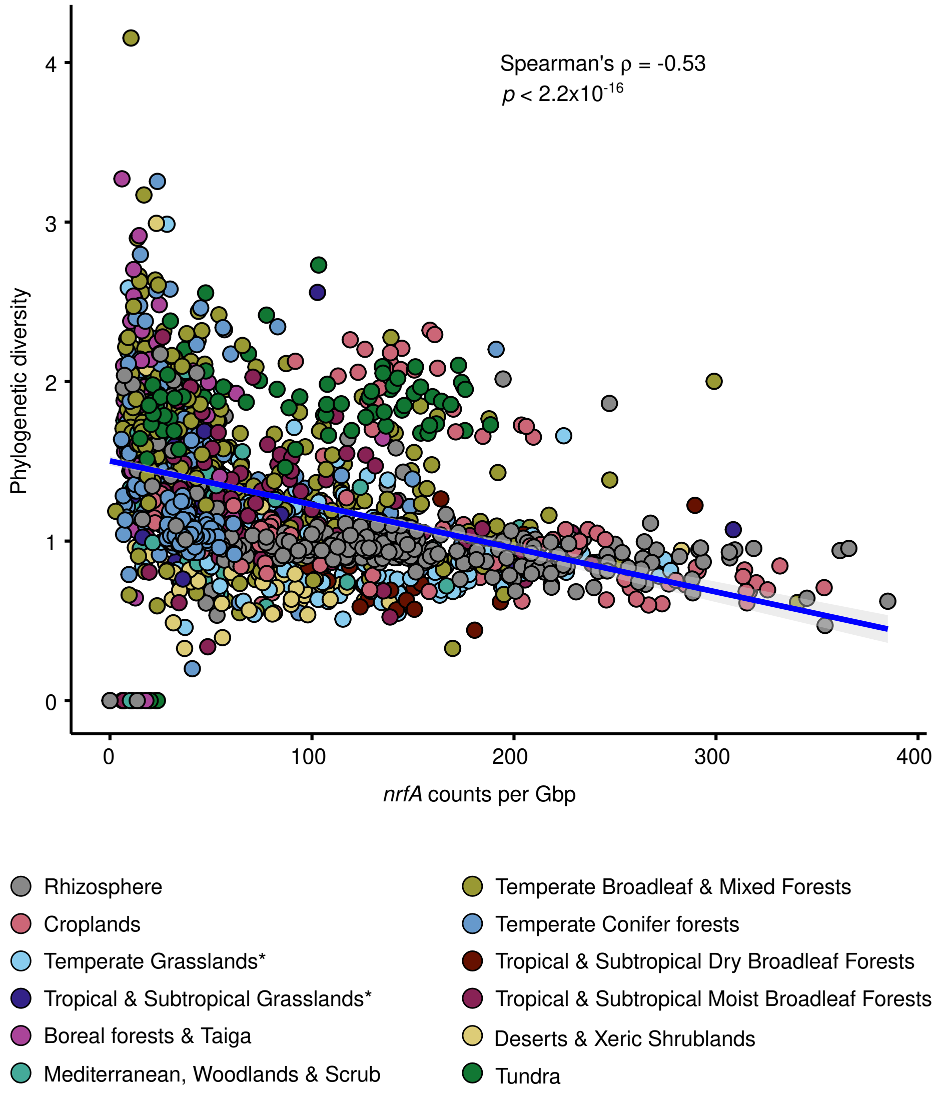


**Supplementary Figure 3. Correlation plot between normalized *nrfA* counts and phylogenetic diversity in soil and rhizosphere metagenomes.** Normalized *nrfA* counts were calculated as the ratio between *nrfA* counts and the total number of base pairs sequenced in each metagenome. Phylogenetic diversity was normalized by the number of *nrfA* counts in each sample. *The biome name also includes savannas and shrublands.

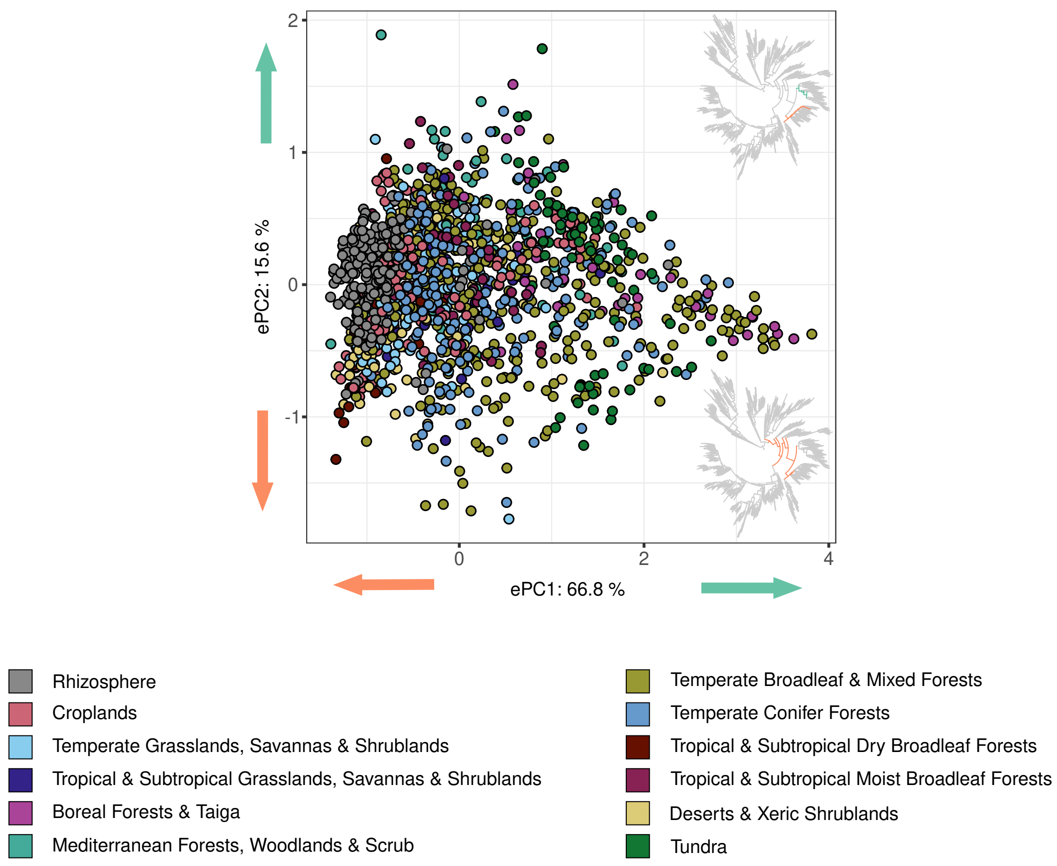


**Supplementary Figure 4. Phylogeny-based community composition across biomes.** Edge principal component analysis showing differences in *nrfA* community composition between metagenomes grouped into biomes. Inset trees show which lineages are driving the separation of samples in positive (turquoise) and negative (orange) direction along each axis. The ordination was performed on metagenomes with ≥ 20 *nrfA* placements (n = 1,475).

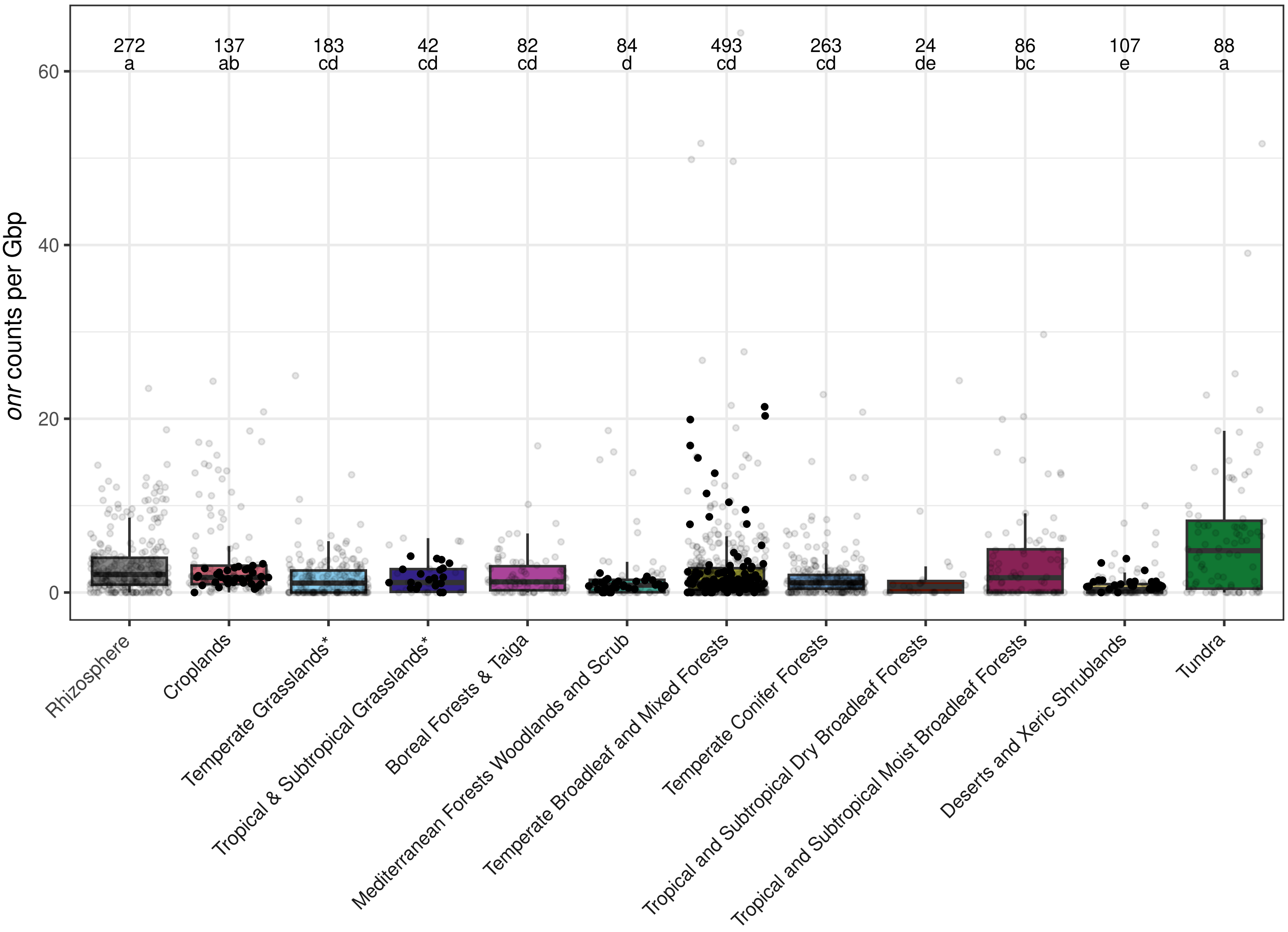


**Supplementary Figure 5. Abundance of *onr* across biomes.** Normalized *onr* counts per biome, calculated as the ratio between *onr* counts and the total number of base pairs (Gbp) sequenced in each metagenome. Significant differences are denoted with different letters (Kruskal-Wallis test, $H = 145$, $P < 0.001$), together with the number of metagenomes representing each biome above the boxplots. Boxes are bounded on the first and third quartiles; horizontal lines represent medians. Whiskers denote 1.5× the interquartile range. Data points corresponding to the metagenomes used in the random forest models are shown as filled circles. *The biome name also includes savannas and shrublands.

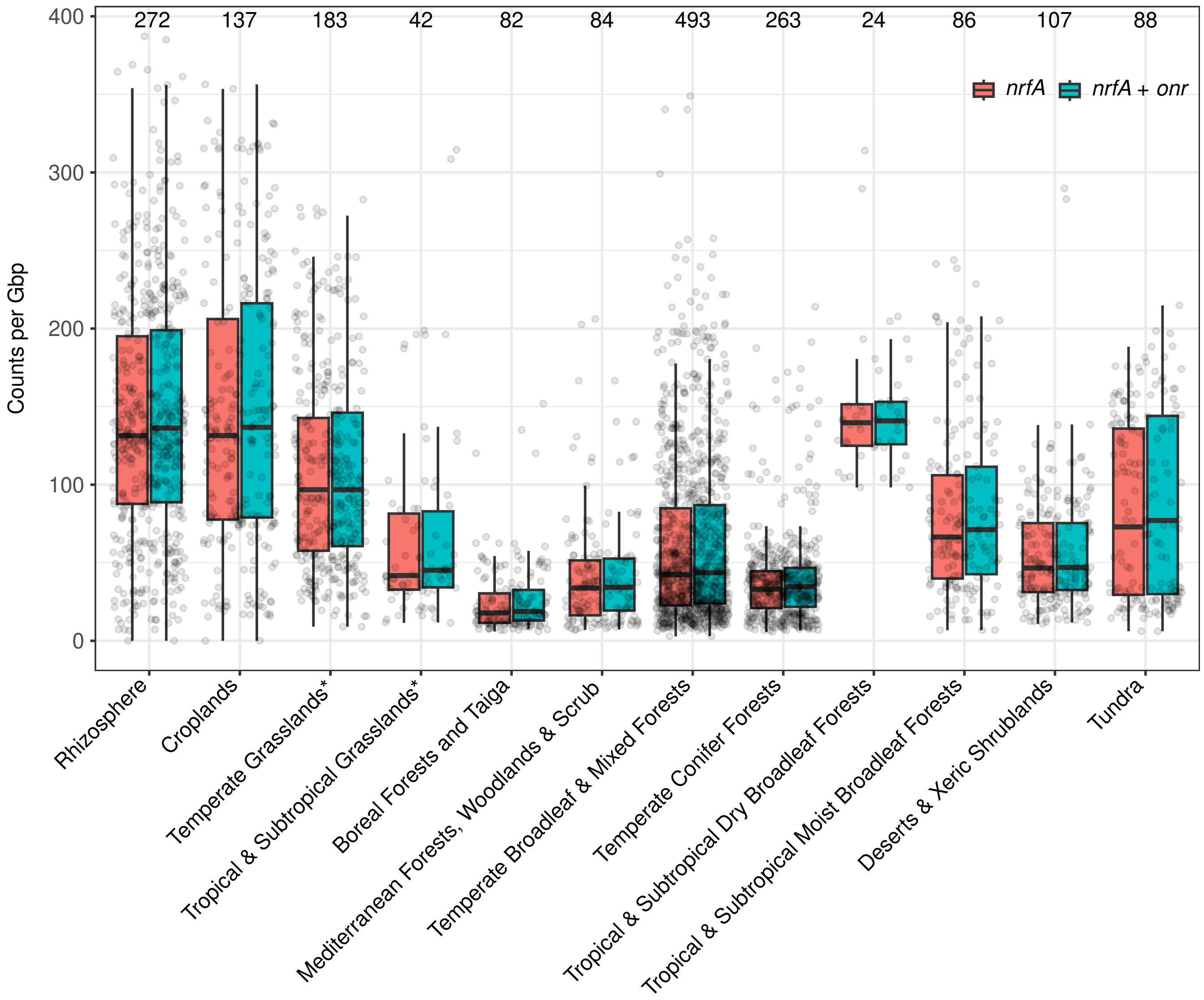


**Supplementary Figure 6. Comparison between *nrfA* and combined *nrfA* and *onr* counts per biome, normalized by the total number of base pairs (Gbp) sequenced in each metagenome.** There was no difference between *nrfA* and *nrfA*+*onr* counts within biomes (number of metagenomes involved in each pair-wise comparison is indicated above the respective boxplots; Wilcoxon-Mann-Withney test, $p > 0.05$). Boxes are bounded on the first and third quartiles; horizontal lines represent medians. Whiskers denote 1.5× the interquartile range.

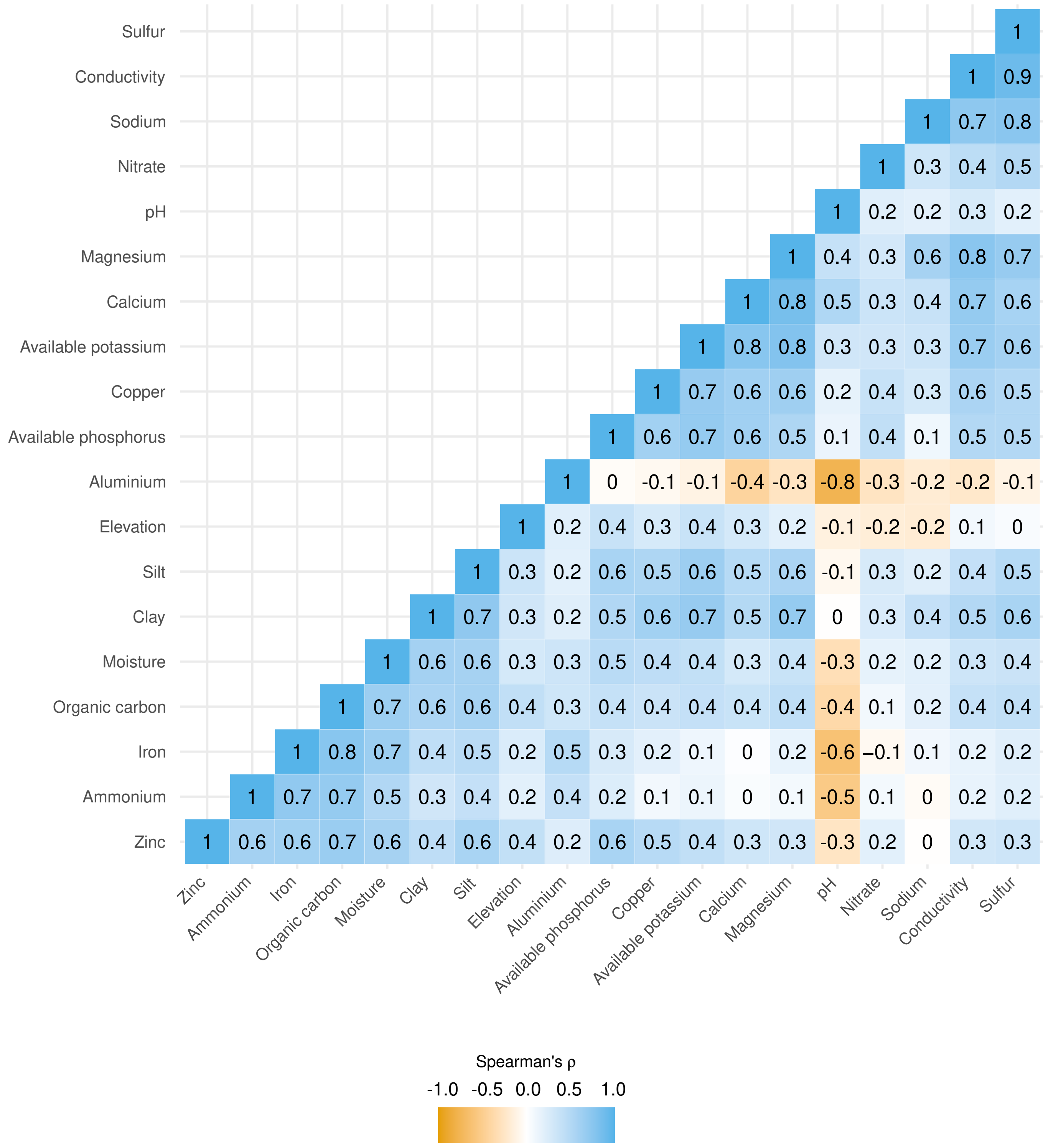


**Supplementary Figure 7. Matrix showing the relationships between environmental variables in the Australian's metagenomes dataset (n = 227), based on Spearman correlations.** See Table 1 for units.

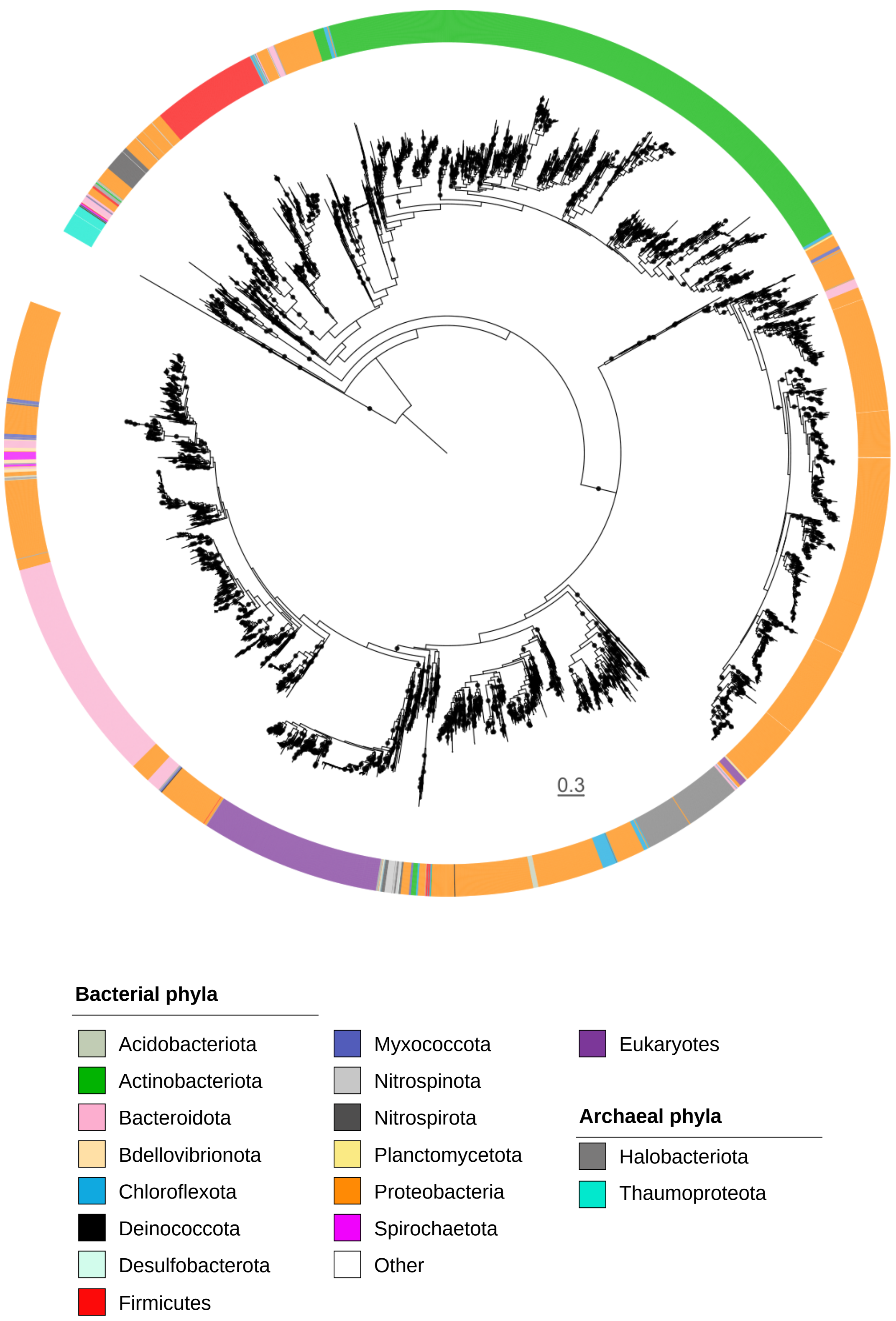


**Supplementary Figure 8. Maximum likelihood phylogeny of 6,422 full-length NirK sequences inferred from the alignment of 573 amino acid positions.** Taxonomic classification at the phylum level is indicated by the color in the ring and is based on the Genome Taxonomy DataBase for archaea and bacteria. Black circles on the phylogeny show support values (SH-aLRT test ≥ 80 % and ultrafast bootstrap ≥ 95 %) and the scale bar denotes the amino acid exchange rate (LG+F+R10). Branch tips corresponding to the multi-copper oxidase outgroup sequences were collapsed (n = 367).

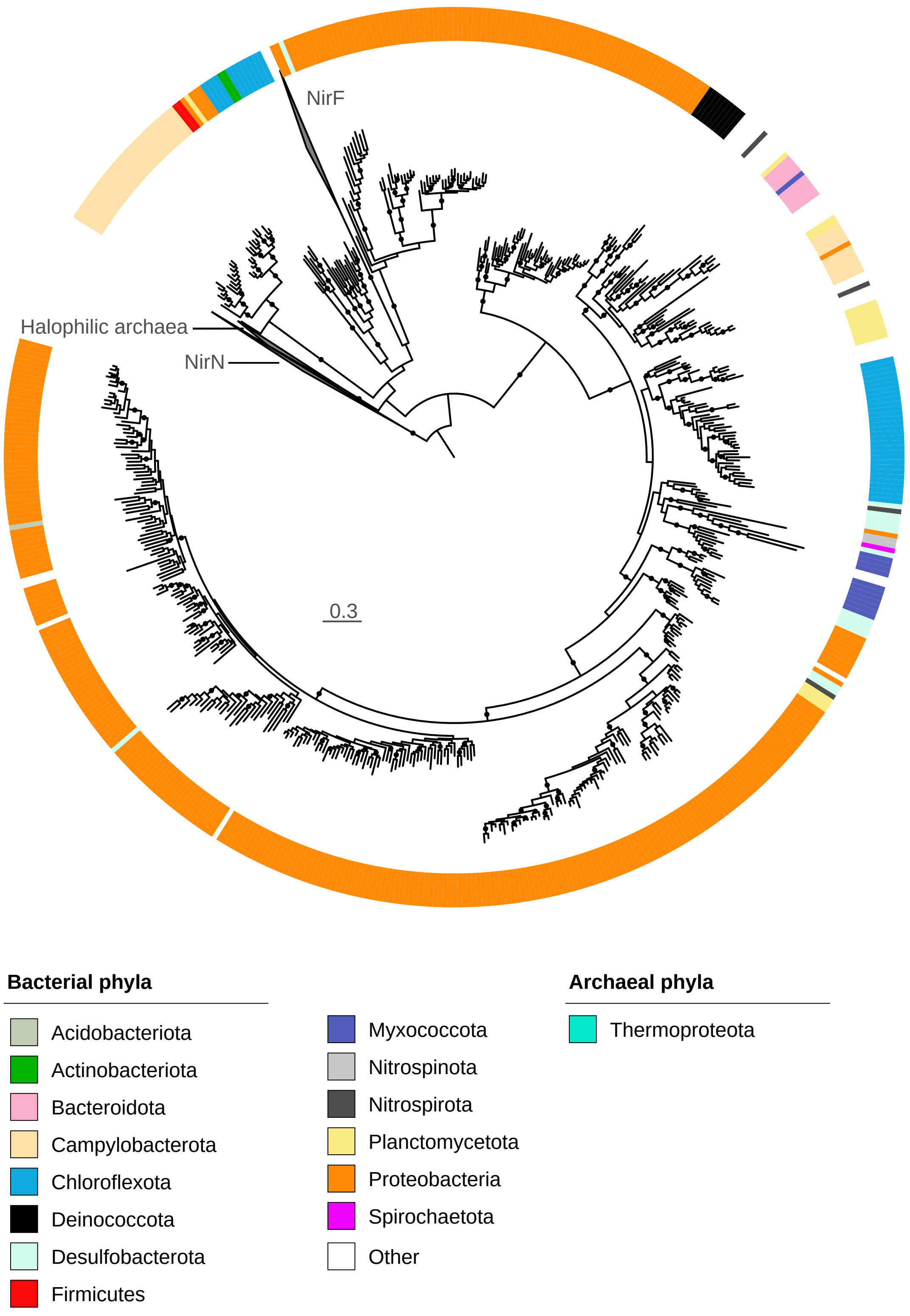


**Supplementary Figure 9. Maximum likelihood phylogeny of 540 full-length NirS sequences inferred from the alignment of 471 amino acid positions.** Taxonomic classification at the phylum level is indicated by the color in the ring and is based on the Genome Taxonomy DataBase. Black circles on the phylogeny show support values (SH-aLRT test ≥ 80 % and ultrafast bootstrap ≥ 95 %) and the scale bar denotes the amino acid exchange rate (LG+F+R9). Branch tips corresponding to the outgroup sequences were collapsed (n = 16 for NirN, n = 13 for NirF and n = 12 for halophilic archaeal NirS-like sequences).

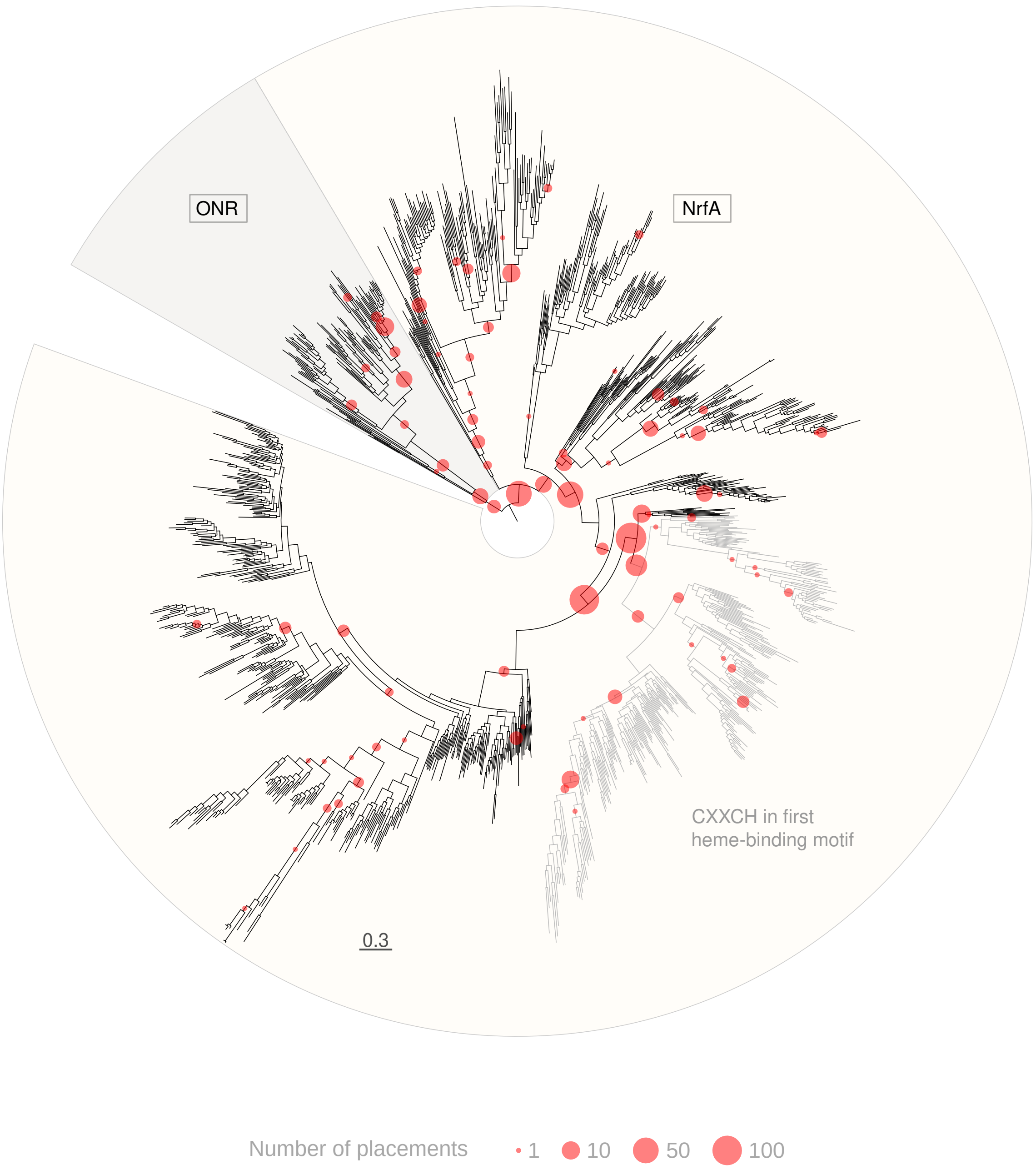


**Supplementary Figure 10. Phylogenetic placement of 86 shredded (50 amino acids) NrfA and ONR homologs on the phylogeny (Cyt c554, HAO, HDH, ihOCC, OTR, MccA, OcwA and OmhA).** Only 6 % of the fragments were placed in the tree (out of 10,000). The scale bar denotes the amino acid exchange rate (WAG+R10). The tree was inferred from the alignment of 350 amino acid positions.

**Supplementary references**


1. Bissett, A. *et al.* Introducing BASE: The Biomes of Australian Soil Environments soil microbial diversity database. *GigaScience* **5**, s13742-016-0126-5 (2016).
2. Hartman, W. H., Ye, R., Horwath, W. R. & Tringe, S. G. A genomic perspective on stoichiometric regulation of soil carbon cycling. *ISME J.* **11**, 2652–2665 (2017).
3. Mendes, L. W., Raaijmakers, J. M., de Hollander, M., Mendes, R. & Tsai, S. M. Influence of resistance breeding in common bean on rhizosphere microbiome composition and function. *ISME J.* **12**, 212–224 (2018).
4. Orellana, L. H., Chee-Sanford, J. C., Sanford, R. A., Löffler, F. E. & Konstantinidis, K. T. Year-round shotgun metagenomes reveal stable microbial communities in agricultural soils and novel ammonia oxidizers responding to fertilization. *Appl. Environ. Microbiol.* **84**, e01646-17 (2018).
5. Xu, J. *et al.* The structure and function of the global citrus rhizosphere microbiome. *Nat. Commun.* **9**, 4894 (2018).
6. Bahram, M. *et al.* Structure and function of the global topsoil microbiome. *Nature* **560**, 233–237 (2018).
7. Wilhelm, R. C. *et al.* A metagenomic survey of forest soil microbial communities more than a decade after timber harvesting. *Sci. Data* **4**, 170092 (2017).
8. Sorensen, J. W., Dunivin, T. K., Tobin, T. C. & Shade, A. Ecological selection for small microbial genomes along a temperate-to-thermal soil gradient. *Nat. Microbiol.* **4**, 55–61 (2019).
9. Diamond, S. *et al.* Mediterranean grassland soil C–N compound turnover is dependent on rainfall and depth, and is mediated by genomically divergent microorganisms. *Nat. Microbiol.* **4**, 1356–1367 (2019).
10. Woodcroft, B. J. *et al.* Genome-centric view of carbon processing in thawing permafrost. *Nature* **560**, 49–54 (2018).
11. Bandla, A., Pavagadhi, S., Sridhar Sudarshan, A., Poh, M. C. H. & Swarup, S. 910 metagenome-assembled genomes from the phytobiomes of three urban-farmed leafy Asian greens. *Sci. Data* **7**, 278 (2020).
12. Levy, A. *et al.* Genomic features of bacterial adaptation to plants. *Nat. Genet.* **50**, 138–150 (2018).
13. Crovadore, J. *et al.* Metagenomes of soils samples from an established perennial cropping system of asparagus treated with biostimulants in southern France. *Genome Announc.* **5**, 00511–17 (2017).
14. Molina-Montenegro, M. A. *et al.* A first insight into the structure and function of rhizosphere microbiota in Antarctic plants using shotgun metagenomic. *Polar Biol.* **42**, 1825–1835 (2019).
15. Singh, R. P., Johri, A. K. & Dua, M. Metagenomic analysis of microbial diversity in cotton rhizosphere soil in Alwar, India. *Microbiol. Resour. Announc.* **9**, 00987–20 (2020).
16. Blair, P. M. *et al.* Exploration of the biosynthetic potential of the Populus microbiome. *mSystems* **3**, 00045–18 (2018).
17. Babalola, O. O., Alawiye, T. T., Lopez, C. R. & Ayangbenro, A. S. Shotgun metagenomic sequencing data of sunflower rhizosphere microbial community in South Africa. *Data Brief* **31**, 105831 (2020).
