# Supplementary material for: Phyloecology of nitrate ammonifiers and their importance relative to denitrifiers in global terrestrial biomes
Source: Nat Commun. 2023 Dec 12;14:8249. doi: 10.1038/s41467-023-44022-3 (PMC10716430; doi:10.1038/s41467-023-44022-3)
Supplement: Supplementary file 4 — Reporting Summary [file 41467_2023_44022_MOESM4_ESM.pdf]

nature portfolio

Corresponding author(s): Sara Hallin

Last updated by author(s): Oct 20, 2023

Reporting Summary

Nature Portfolio wishes to improve the reproducibility of the work that we publish. This form provides structure for consistency and transparency in reporting. For further information on Nature Portfolio policies, see our Editorial Policies and the Editorial Policy Checklist.

Statistics

For all statistical analyses, confirm that the following items are present in the figure legend, table legend, main text, or Methods section.

n/a	Confirmed	
☐	☒	The exact sample size (*n*) for each experimental group/condition, given as a discrete number and unit of measurement
☒	☐	A statement on whether measurements were taken from distinct samples or whether the same sample was measured repeatedly
☐	☒	The statistical test(s) used AND whether they are one- or two-sided<br>*Only common tests should be described solely by name; describe more complex techniques in the Methods section.*
☐	☒	A description of all covariates tested
☐	☒	A description of any assumptions or corrections, such as tests of normality and adjustment for multiple comparisons
☐	☒	A full description of the statistical parameters including central tendency (e.g. means) or other basic estimates (e.g. regression coefficient) AND variation (e.g. standard deviation) or associated estimates of uncertainty (e.g. confidence intervals)
☐	☒	For null hypothesis testing, the test statistic (e.g. *F*, *t*, *r*) with confidence intervals, effect sizes, degrees of freedom and *P* value noted<br>*Give P values as exact values whenever suitable.*
☒	☐	For Bayesian analysis, information on the choice of priors and Markov chain Monte Carlo settings
☒	☐	For hierarchical and complex designs, identification of the appropriate level for tests and full reporting of outcomes
☒	☐	Estimates of effect sizes (e.g. Cohen's *d*, Pearson's *r*), indicating how they were calculated

Our web collection on statistics for biologists contains articles on many of the points above.

Software and code

Policy information about availability of computer code

Data collection	No software was used for data collection
Data analysis	HMMER v. 3.2, ARB v. 7.0, CD-HIT v. 4.8.1, FastTreeMP v. 2.1.11, IQ-TREE v. 2.1.3, iTOL v5, Busco v. 5.2.2, GTDB-tk v. 1.5.0, GraftM v. 0.13.1, RaxML v. 7.7.2, Grinder 0.5.4, Gappa v. 0.8.1, Guppy v. 1.1. R (v. 4.2.0) packages: sp v. 1.4-6, rgeos v.0.5-5, rgdal v. 1.5-23, ggspatial v. 1.1.5, rnaturalearth v. 0.1.0, rnaturalearthdata v. 0.1.0, rgeos v.0.5-5 and sf v. 1.0-7, agricolae v. 1.3.5, VSURF v. 1.1.0, randomForest v. 4.7–1 , iml v. 0.9.0.<br>The bash and R scripts used in this study are available in Zenodo (DOI: 10.5281/zenodo.8026657).

For manuscripts utilizing custom algorithms or software that are central to the research but not yet described in published literature, software must be made available to editors and reviewers. We strongly encourage code deposition in a community repository (e.g. GitHub). See the Nature Portfolio guidelines for submitting code & software for further information.

Data

Policy information about availability of data

All manuscripts must include a data availability statement. This statement should provide the following information, where applicable:

- Accession codes, unique identifiers, or web links for publicly available datasets
- A description of any restrictions on data availability
- For clinical datasets or third party data, please ensure that the statement adheres to our policy

The metadata, sequence alignments, HMM models and phylogenetic trees (newick format) generated in this study have been deposited in Zenodo (https://doi.org/10.5281/zenodo.8026657). All genome assemblies and metagenomes used in this study were publicly available and their accession codes are provided in Zenodo. The alignment for Nor was kindly provided by Ranjani Murali. The Genome Taxonomy database can be accessed at: https://gtdb.ecogenomic.org/.

Research involving human participants, their data, or biological material

Policy information about studies with human participants or human data. See also policy information about sex, gender (identity/presentation), and sexual orientation and race, ethnicity and racism.

Reporting on sex and gender	N/A
Reporting on race, ethnicity, or other socially relevant groupings	N/A
Population characteristics	N/A
Recruitment	N/A
Ethics oversight	N/A

Note that full information on the approval of the study protocol must also be provided in the manuscript.

Field-specific reporting

Please select the one below that is the best fit for your research. If you are not sure, read the appropriate sections before making your selection.

☐ Life sciences ☐ Behavioural & social sciences ☒ Ecological, evolutionary & environmental sciences

For a reference copy of the document with all sections, see nature.com/documents/nr-reporting-summary-flat.pdf

Ecological, evolutionary & environmental sciences study design

All studies must disclose on these points even when the disclosure is negative.

Study description	We constructed phylogenies of nirS/nirK/nrfA by screening genome assemblies publicly available on GenBank and recruited gene fragments in 1,861 metagenomes covering the major terrestrial biomes to assess the relative importance and drivers of these two processes.
Research sample	We used publicly available genome assemblies and metagenomes. They were downloaded from NCBI (https://www.ncbi.nlm.nih.gov/), the National Ecological Observatory Network (NEON; https://data.neonscience.org/data-products/DP1.10107.001/RELEASE-2021) and the Biome of Australia Soil Environments database (BASE; https://bioplatforms.com/projects/soil-biodiversity/).
Sampling strategy	We downloaded all archaeal and bacterial genome assemblies available on Genbank in October 2021. We also downloaded soil and rhizosphere metagenomes that were generated using Illumina short-read technology (read length ≥ 150 nt) and consisting of a minimum of 100,000 reads.
Data collection	AS, GP and CMJ downloaded the data from public servers between 2019 and 2021.
Timing and spatial scale	N/A
Data exclusions	Metagenomes with read length < 150 nt and consisting of less than 100,000 reads were excluded.
Reproducibility	This study does not involve experimental work. The code and data necessary to repeat the analyses are provided in Zenodo.
Randomization	N/A
Blinding	N/A

Did the study involve field work? ☐ Yes ☒ No

Reporting for specific materials, systems and methods

We require information from authors about some types of materials, experimental systems and methods used in many studies. Here, indicate whether each material, system or method listed is relevant to your study. If you are not sure if a list item applies to your research, read the appropriate section before selecting a response.

Materials & experimental systems

n/a	Involved in the study	
☒	☐	Antibodies
☒	☐	Eukaryotic cell lines
☒	☐	Palaeontology and archaeology
☒	☐	Animals and other organisms
☒	☐	Clinical data
☒	☐	Dual use research of concern
☒	☐	Plants

Methods

n/a	Involved in the study	
☒	☐	ChIP-seq
☒	☐	Flow cytometry
☒	☐	MRI-based neuroimaging
